# Microstate connectivity alterations in patients with early Alzheimer’s disease

**DOI:** 10.1186/s13195-015-0163-9

**Published:** 2015-12-31

**Authors:** Florian Hatz, Martin Hardmeier, Nina Benz, Michael Ehrensperger, Ute Gschwandtner, Stephan Rüegg, Christian Schindler, Andreas U. Monsch, Peter Fuhr

**Affiliations:** Department of Neurology, University Hospital of Basel, Petersgraben 4, 4031 Basel, Switzerland; Memory Clinic, University Center for Medicine of Aging Basel, Felix Platter Hospital, Basel, Switzerland; Swiss Tropical and Public Health Institute, University of Basel, Basel, Switzerland

## Abstract

**Introduction:**

Electroencephalography (EEG) microstates and brain network are altered in patients with Alzheimer’s disease (AD) and discussed as potential biomarkers for AD. Microstates correspond to defined states of brain activity, and their connectivity patterns may change accordingly. Little is known about alteration of connectivity in microstates, especially in patients with amnestic mild cognitive impairment with stable or improving cognition within 30 months (aMCI).

**Methods:**

Thirty-five outpatients with aMCI or mild dementia (mean age 77 ± 7 years, 47 % male, Mini Mental State Examination score ≥24) had comprehensive neuropsychological and clinical examinations. Subjects with cognitive decline over 30 months were allocated to the AD group, subjects with stable or improving cognition to the MCI-stable group. Results of neuropsychological testing at baseline were summarized in six domain scores. Resting state EEG was recorded with 256 electrodes and analyzed using TAPEEG. Five microstates were defined and individual data fitted. After phase transformation, the phase lag index (PLI) was calculated for the five microstates in every subject. Networks were reduced to 22 nodes for statistical analysis.

**Results:**

The domain score for verbal learning and memory and the microstate segmented PLI between the left centro-lateral and parieto-occipital regions in the theta band at baseline differentiated significantly between the groups. In the present sample, they separated in a logistic regression model with a 100 % positive predictive value, 60 % negative predictive value, 100 % specificity and 77 % sensitivity between AD and MCI-stable.

**Conclusions:**

Combining neuropsychological and quantitative EEG test results allows differentiation between subjects with aMCI remaining stable and subjects with aMCI deteriorating over 30 months.

**Electronic supplementary material:**

The online version of this article (doi:10.1186/s13195-015-0163-9) contains supplementary material, which is available to authorized users.

## Introduction

In the United Kingdom cognitive decline affects approximately 18 % in the elderly [1], and early classification of the underlying pathology and prognosis is difficult. As personalized medicine may gain importance in the future, the distinction between patients with a prodromal syndrome of neurodegenerative dementia and patients with other reasons for cognitive impairment, such as as depressive and sleep disorders or neurovascular diseases, becomes relevant. For early identification of dementia, the term *mild cognitive impairment* (MCI) was defined as a potential prodromal syndrome without significant impairment in activities of daily living. This term was replaced with *mild neurocognitive disorder* in the revised *Diagnostic and Statistical Manual of Mental Disorders, Fifth Edition*, criteria [2]. The term is unspecific as the disorder can be caused by various pathologies [3], and a considerable number of patients with MCI remain stable or improve over time [4]. Diagnostic criteria for MCI due to Alzheimer’s disease were established [5, 6]. The rate of progression to AD in patients with MCI varies, depending on study design and definition of MCI. Maximally, 40 % of patients with amnestic (single or multiple domains) mild cognitive impairment (aMCI) at baseline progress to dementia within 2–3 years [7, 8]. However, novel treatment strategies require initiation of treatment at the earliest possible time [9]; therefore, the corroboration of the diagnosis and the identification of the cause of MCI are important.

Quantitative electroencephalography (qEEG) is increasingly being used to characterize cognitive impairment in different disorders [10–12]. However, it is unknown whether qEEG reliably identifies patients at an early stage of AD with clear progression of neuropsychological deficits and/or progression to dementia within the following years.

The aim of the present study was to find reliable qEEG biomarkers for the identification of patients with early cognitive deficits and at high risk of considerable cognitive decline and/or progression to AD dementia. The study included frequency analysis in signal space and a combined microstate and connectivity analysis. On the basis of the literature, patients with MCI progressing to AD are expected to show an increased connectivity in the theta band while the connectivity in the alpha and beta bands is decreased. Moreover, we hypothesize that alteration in connectivity in microstates correlates disparately with neuropsychological domain scores.

## Methods

### Patients

Thirty-five outpatients (Table 2) with either aMCI (*n* = 12) or mild AD (Mini Mental State Examination [MMSE] [13] score ≥24/30; *n* = 23) attending the Memory Clinic, University Center for Medicine of Aging Basel, Felix Platter-Hospital, Basel, Switzerland, participated in the study. aMCI was diagnosed according to the definition of Winblad et al. [3]. Probable AD was diagnosed according to the definition of McKhann et al. [14]. Exclusion criteria consisted of MMSE score <24/30, any significant diagnosis other than AD, and antiepileptic or antipsychotic drug treatment influencing electroencephalography (EEG) recordings. All patients taking benzodiazepines were either excluded or had their treatment stopped at least 48 h before EEG recording. Twenty-one patients had a clinical follow-up examination at about 30 months (mean observation time 30.1 ± 1.9 months, range 29–31 months), along with a reevaluation of their diagnoses. Among the patients with an initial diagnosis of probable AD, nine patients had a clinical examination and eight patients a standardized telephone visit at 30 months. For six patients, only a follow-up visit at 15 months was available. Yet, all 23 patients showed a decline in cognition over time. All 12 patients with an initial diagnosis of aMCI had a clinical follow-up visit at 30 months, and 9 patients had mild cognitive impairment with stable or improving cognition within 30 months (MCI-stable). Three patients with aMCI had deteriorated and were thus allocated for analysis to the AD group (*n* = 26).

For comparison purposes, a group of 26 cognitively healthy control (HC) subjects was frequency-matched to the AD group according to sex, age, and education (Table 2). Inclusion criteria for HC subjects were a subjective report of good health and neuropsychological examination results within normal limits (i.e., *z*-scores greater than or equal to −1.28). Exclusion criteria were a past and/or current diagnosis of any major brain disorder, alcoholism, psychiatric disorder, and general anesthesia within the previous 3 months. The study was approved by the local ethics committee (Ethikkomission beider Basel reference number 260/09). Written informed consent was obtained from all participants.

### Neuropsychological assessments

Raw scores derived from a comprehensive neuropsychological assessment battery were transformed into demographically (age, sex, and education level) adjusted *z*-scores [15]. Six domain scores were created according to Table 4 in the article by Beck et al. [16]. Briefly, these domains were assessed using the following tests: (1) for verbal attention, the digit span (forward and backward) from the German version of the Wechsler Memory Scale [17]; (2) for visual attention, the Corsi block-tapping test (forward and backward) from the German version of the Wechsler Memory Scale [17]; (3) for verbal learning memory, either the Consortium to Establish a Registry for Alzheimer’s Disease Neuropsychological Assessment Battery (CERAD-NAB [18]; *n* = 13) or the German version of the California Verbal Learning Test [19] (*n* = 22); (4) for visual learning and memory, either the CERAD-NAB figures [18] or the Rey-Osterrieth complex figure test [20]; (5) for verbal language production, the 15-item Boston Naming Test [18], animal fluency [21], and phonemic fluency (s-words) [22]; and (6) for executive motor ability, the Trail Making Test [23] and the Five-Point Test [24]. Executive visual ability could not be evaluated, because the Stroop effect test was not administered to the HC subjects.

### EEG recording

EEG was recorded with a 256-channel EEG system (Geodesic EEG System 300, DC-amplifier, sampling rate 1000 Hz, high-pass filter 0.01 Hz, vertex reference, impedance ≤40 kΩ; Electrical Geodesics Inc. [EGI], Eugene, OR USA). Subjects were instructed to relax but to stay awake and to minimize eye and body movements. A continuous EEG with the subject’s eyes closed was recorded for 12 minutes. During data acquisition, a technician monitored a subset of electrodes online to check for vigilance and artifacts.

### EEG preprocessing

A fully automated preprocessing procedure was carried out using Toolbox for Automated Processing of EEG (TAPEEG) v2.5 software (https://sites.google.com/site/tapeeg/) [25]. Briefly, segments of 25–200 seconds containing the least amount of artifacts and sleepiness were automatically selected. Data of 214 electrodes (excluding cheek and neck electrodes) were filtered (0.5–70 Hz, high-order least-squares filter), and bad channels were automatically detected by FASTER and FieldTrip algorithms [26, 27]. Using independent component analysis (EEGLAB [28]), components loading the electrocardiogram, line noise in single electrodes or single gross artifacts were excluded (at maximum 5 % of components). For epoch selection, the EEG was re-referenced to average reference, bad channels were interpolated using spherical splines [29], and a combined segment at least 180 seconds in length was created.

### Spectral EEG analysis

Spectral analysis has been described elsewhere [30]. Twelve epochs of 4 seconds were automatically selected. Power spectra were calculated at each electrode (Thomson multitaper method), and at every electrode the median spectrum of the 12 epochs was determined. Spectral analysis was done on a regional level of spatial resolution based on 22 anatomically defined regions comprising 7 or 8 electrodes (*n* = 170, excluding electrodes in the midline and at the outer border) (see Additional file 1: Figure S1). From the median spectrum of each brain region, relative band power was calculated in five frequency bands (delta 1–4 Hz, theta 4–8 Hz, alpha1 8–10 Hz, alpha2 10–13 Hz, beta 13–30 Hz). Relative band power was defined as the absolute band power in a single frequency band divided by the band power 1–30 Hz. For statistical analysis, regional power was logit-transformed [31]. Peak and median frequency were determined at all parieto-occipital electrodes, and the median of the peak and median frequency were used for further analysis.

### Microstate segmentation

The global field power (GFP) was calculated as the standard deviation of the data at each time point [32, 33]:

$$ GF{P}_t=\sqrt{\frac{{\displaystyle {\sum}_{i=1}^n}{\mathrm{u}}_i^2}{n}}\kern1.25em \left(\mathrm{n} = \mathrm{number}\ \mathrm{of}\ \mathrm{channels},\ \mathrm{u} = \mathrm{amplitude}\ \mathrm{in}\ \mathrm{u}\mathrm{V}\ \mathrm{at}\ \mathrm{t}\mathrm{ime}\ \mathrm{point}\ \mathrm{t}\right) $$

Only time points of local maxima of GFP were selected, and a *k*-means clustering with squared correlation as distance measure was used to obtain the most representative topographies. *k*-Means clustering was calculated for results with 2–20 clusters, and the optimal number of clusters was defined based on the Krzanowski-Lai criterion using an L-curve via an adaptive pruning algorithm [34], resulting in five different microstates as optimum. By fitting the individual data to the five template microstates using a temporal smoothing with a window size of 12 milliseconds [35], a vector was created for every subject, competitively labeling every time point to one of the five microstate classes.

### Functional connectivity

Phase lag index (PLI) measures were first calculated using a standard approach. Data were filtered using a Butterworth filter to four predefined frequency bands (theta 4–8 Hz, alpha1 8–10 Hz, alpha2 10–13 Hz, beta 13–30 Hz). Phase estimation was archived using a Hilbert transformation. The phase difference distribution was obtained from a time series (*t*_1_, …, *t*_k_) of phase differences (ΔΦ) between two signals, and the asymmetry of the phase difference distribution was calculated as described by Stam et al. [36]:

$$ PLI = \frac{1}{k}\left|{\displaystyle \sum_{i=1}^k} sign\left[ \sin \left(\varDelta \phi (i)\right)\right]\right|\kern0.5em \left(\mathrm{k} = \mathrm{number}\ \mathrm{of}\ \mathrm{time}\ \mathrm{points},\ \varDelta \phi = \mathrm{phase}\ \mathrm{differences}\ \mathrm{between}\ \mathrm{two}\ \mathrm{channels}\right) $$

PLI was calculated using 12 epochs of 4 seconds and averaging the resulting matrix into 1 matrix per subject and frequency band. In the second approach, the microstate segmented phase lag index (msPLI) was calculated. For the calculation of msPLI, the Hilbert transformation was applied to the full-length EEG using a sliding window of 4 seconds with a 50 % Hanning window. For every microstate class, 4 stitched periods each of 4000 phase differences (4 seconds) were then extracted using the time frames indicated by the microstate label vector. The number of four epochs per microstate, subject, and frequency band was selected, as this minimal amount of epochs per microstate was available in almost all EEGs, given the recording time of EEG data. Like in the standard approach, the 20 resulting matrices (4 epochs × 5 microstates) were averaged per subject. For statistical analysis, electrodes were grouped into 22 regions of interest [25], comprising 11 regions per hemisphere, excluding electrodes in the midline, neck, and face.

### Graph measures

Graph measures were calculated based on the full average msPLI weight matrix per subject in each frequency band (*n* = 214 nodes). To avoid arbitrary thresholds and unconnected nodes, weighted network analysis was employed in which each link was equivalent to the measured PLI of two interconnected nodes. Graph analysis results were calculated according to Table 1. The respective formulas were implemented in TAPEEG.

**Table 1:** Graph measures as calculated for each subject

Name		Formula	Reference
degree		= mean(W) (all links of a single node)	
Average clustering coefficient	Cw	*Cw* = *mean*(*C*)	[38]
		$$ {C}_i = \frac{\underset{l\ne k}{{\displaystyle {\sum}_{k\ne i}}{\displaystyle {\sum}_{l\ne i}}{W}_{ik}{W}_{il}{W}_{kl}}}{\underset{i\ne k}{{\displaystyle {\sum}_{k\ne i}}{\displaystyle {\sum}_{l\ne i}}{W}_{ik}{W}_{il}}} $$	
Normalized clustering coefficient	Gamma	Cw / Cr	[49]
		(Cr = average of 50 randomized input matrices)	
Average path length	Lw	$$ L = \raisebox{1ex}{$1$}\!\left/ \!\raisebox{-1ex}{$W$}\right. $$	[38]
		*L* = ∞ (*if W* = 0)	
		$$ Lw = \frac{1}{\frac{1}{N\left(N-1\right)}*\ {\displaystyle {\sum}_{i=1}^N}{\displaystyle {\sum}_{j\ne i}^N}\left(1/{L}_{ij}\right)} $$	
Normalized average path length	Lambda	Lw / Lr	[49]
		(Lr = average of 50 randomized matrices)	
Degree correlation	Rw	Rw = Pearson correlation of degrees of pairs of neighbors	[50]
Degree diversity	Kw	$Kw=\frac{\left\langle degre{e}^2\right\rangle }{\left\langle degre e\right\rangle }$	[44]
Radius	Radius	Ec = maximal shortest path of single node	[51]
		Radius = = min (Ec)	
Diameter	Diameter	= max(Ec)	

The weighted clustering coefficient *C* quantifies the intensities of the subgraphs of a node and is equivalent to the unweighted clustering coefficient normalized by the average intensities of triangles at the node if the weight matrix is symmetric and weights range between 0 and 1 [37]. The average overall *C* is the mean clustering coefficient (*C*w), a global measure of functional segregation of the network [38, 39]. The weighted shortest path length *L* gives the average of the shortest distances of one node to each other node in the network, where shortest distance in the weighted case is defined as the smallest inverse of the sum of PLI values of connecting edges. The average overall *L* is the weighted average path length (*L*w), a global measure of functional integration of the network [39, 40]. To make graph measures independent of network size and achieve better comparability between subjects, the measures were normalized [37]. Edge weights of an original network were randomly reshuffled, preserving network size but destroying network structure, and *C*w and *L*w were calculated for this random network. Using the average *C*w and *L*w of 50 surrogate random networks iterated five times in the denominator and *C*w and *L*w in the nominator, the normalized *C*w or gamma and the normalized *L*w or lambda were calculated. The degree diversity (*K*w) represents the distribution of degrees in a network. The degree is the mean connectivity of a single node to all other nodes. A higher *K*w stands for a network with only a few highly connected nodes, also called *hubs* [41]. The degree correlation expresses the amount of interconnection between nodes with similar degrees. This measure is related to the concept of a “rich club” [42], whereas nodes with higher degrees are preferentially interconnected. The distance matrix of a graph comprises all pairwise distances. Its maximum corresponds to the graph diameter, its minimum to the graph radius [41].

### Statistics

Demographic characteristics were compared between groups using nonparametric tests. qEEG variables, including regional connectivities and results of neuropsychological tests, were compared between the three groups using analysis of variance (ANOVA). Subsequently, post hoc *t* tests between subgroups were applied. In case of regional EEG power analysis, permutation tests (ANOVA/*t* test with 10,000 permutations) were used [43]. A logistic regression analysis with backward elimination to classify groups was performed using the significant EEG measures from permutation tests and the neuropsychological test results at baseline as independent variables. Subsequently a receiver operating characteristic (ROC) analysis was performed. Results with *p* values <0.05 were considered significant. Analyses were done using TAPEEG [25] and IBM SPSS® software (IBM, Armonk, NY, USA).

## Results

### Demographics

No significant differences between the three patient groups regarding age, education, or sex were found (Kruskal-Wallis test) (see Table 2). MMSE scores of patients with AD were significantly lower than those in the HC group (*p* < 0.01).

**Table 2:** Demographic characteristics (median, lower and upper quartiles)

	AD (*n* = 26)	MCI-stable (*n* = 9)	HC (*n* = 26)
Age, yr	78.5 (73–83)	73 (71–75)	77 (70–81)
Education, yr	13 (11–17)	15 (12–18)	12 (11–15)
Female sex, %	42 %	44 %	46 %
MMS score*	27 (25–28)	29 (28–30)	29 (29–30)
Domain scores [20]			
Verbal Attention	−0.26 (−0.89 to 0.36)	−0.58 (−1.2 to 0.57)	0.06 (−0.56 to 0.99)
Attention	−0.92 (−1.32 to 0.34)	−0.5 (−1.28 to 0.09)	−0.04 (−0.9 to 0.34)
Verbal Learning Memory*	−2.59 (−2.98 to 1.73)	−1.71 (−2.18 to 1.08)	−0.01 (−0.48 to 0.57)
Visual Learning Memory*	−1.18 (−2.01 to 0.61)	−0.31 (−1.52 to 0.47)	0.07 (−0.42 to 0.71)
Verbal Language Production*	−0.68 (−1.2 to 0.11)	−0.19 (−0.7 to 0.1)	0.38 (−0.1 to 1.02)
Executive Motor Ability*	−1.19 (−2.67 to 0.32)	0.55 (−0.65 to 0.72)	0.97 (0.13 to 1.92)

*AD* Alzheimer’s disease, *MCIstable* patients with stable or improving cognition over 30 months, *HC* healthy controls, *MMSE* Mini Mental State Examination

*p < 0.05 by Kruskal-Wallis test

### Neuropsychological assessments

**Fig. 1:**
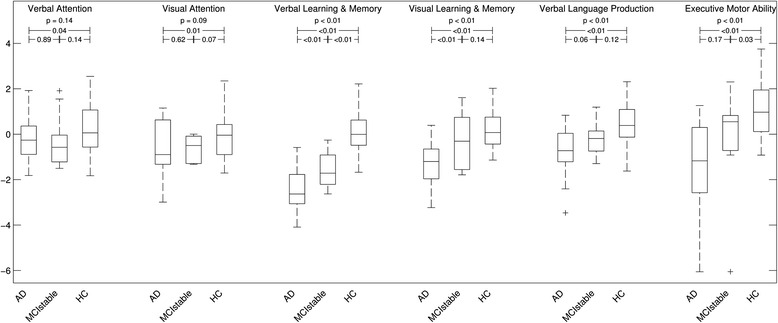
Box plots of domain scores for patients with Alzheimer’s disease (AD), patients with stable or improving cognition within 30 months (MCIstable), and healthy control (HC) subjects. Axes indicate the results of domain *z*-scores. *p* Values shown are the results of analysis of variance and *t* tests

A comparison of the cognitive dimensions scores is shown in Fig. 1. As expected, at baseline, patients with AD performed worse than HC subjects and the group of MCIstable individuals was in between.

### Quantitative EEG

#### Frequency analysis

**Fig. 2:**
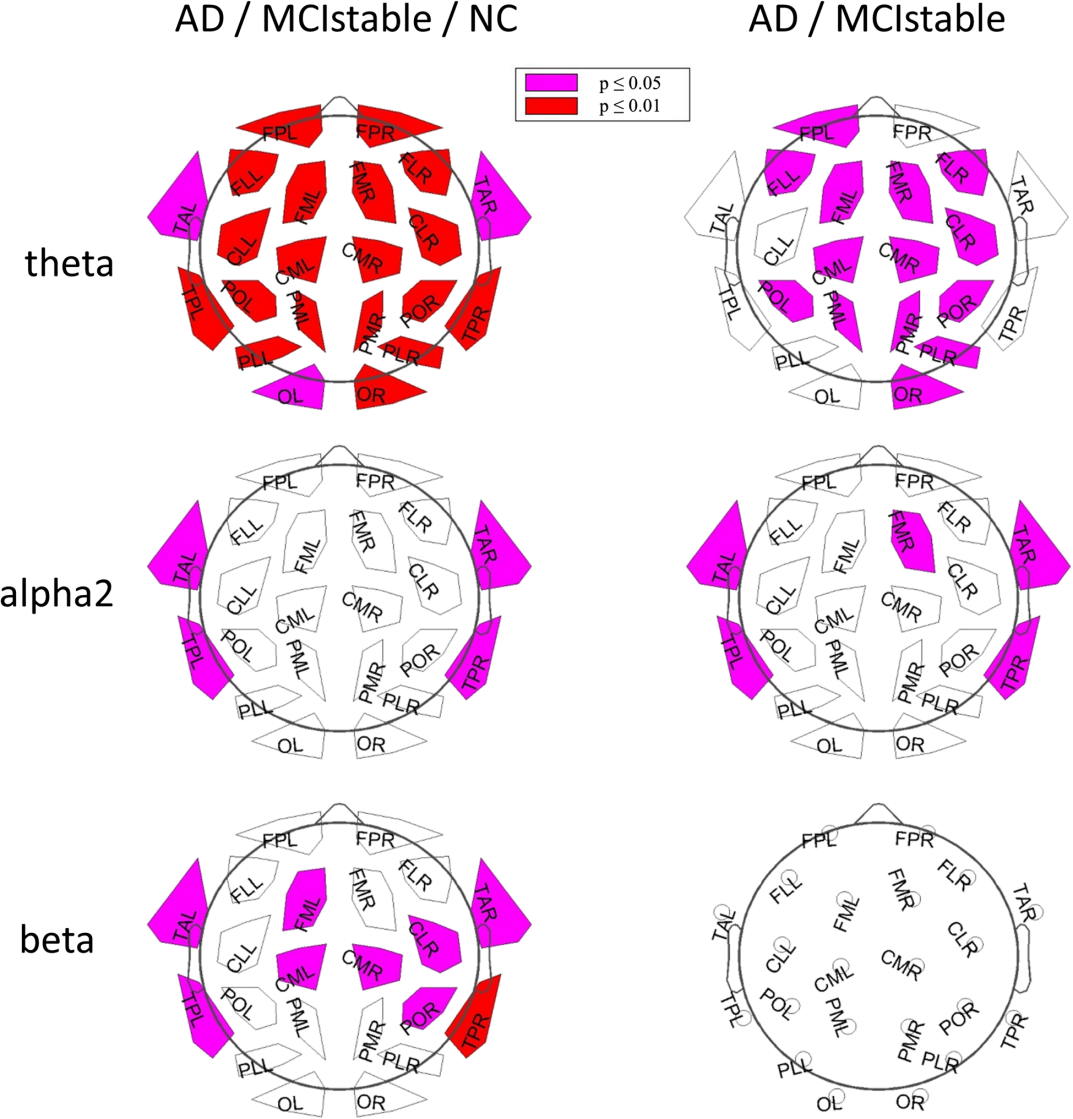
Results of regional power analysis. *FP* frontal parieto-occipital left, *FM* frontal midline, *FL* frontal lateral, *CM* central midline, *CL* central lateral, *TA* temporal anterior, *TP* temporal posterior, *PL* parietal lateral, *PM* parietal midline, *PO* parieto-occipital, *O* occipital, *L* left, *R* right. *Violet*: *p* < 0.05; *red*: *p* < 0.01 (corrected for multiple comparisons), AD = Alzheimer’s disease, MCIstable = patients with stable or improving cognition over 30 months, HC = healthy control subjects

**Fig. 3:**
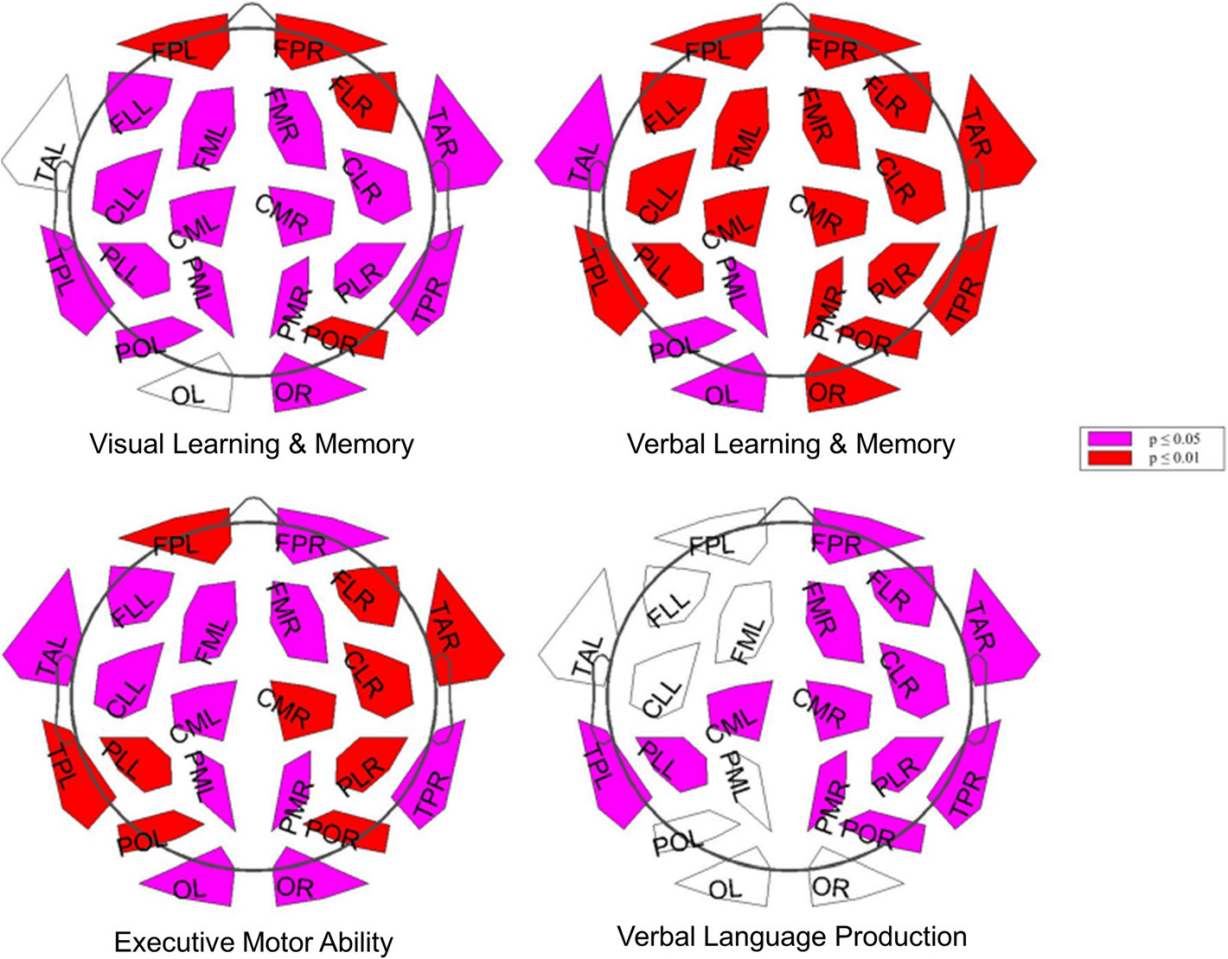
Significant correlations of regional relative theta power and domain scores. *FP* frontal parieto-occipital left, *FM* frontal midline, *FL* frontal lateral, *CM* central midline, *CL* central lateral, *TA* temporal anterior, *TP* temporal posterior, *PL* parietal lateral, *PM* parietal midline, *PO* parieto-occipital, *O* occipital, *L* left, *R* right. *Violet*: *p* < 0.05; *red*: *p* < 0.01 (corrected for multiple comparisons, all subjects included)

HC subjects and MCI-stable patients had significantly lower theta power and higher median frequency than patients with AD and tended to have higher alpha2 power. The results of regional analysis are shown in Fig. 2. Theta power differentiated most significantly between AD and MCI-stable (Table 3), classified with a sensitivity of 67 %, specificity of 85 %, positive predictive value of 88 %, and negative predictive value of 60 %. A higher relative theta power correlated with lower *z*-score for verbal learning and memory, visual learning and memory, executive motor ability, and verbal language production (Fig. 3).

**Table 3:** Global relative power and median frequency

	AD	MCI-stable	HC	*p* value
Delta (1–4 Hz)	0.27 (0.17–0.34)	0.28 (0.23–0.29)	0.27 (0.19–0.32)	n.s.
Theta (4–8 Hz)	0.24 (0.18–0.29)	0.18 (0.14–0.21)	0.15 (0.13–0.21)	<0.01*
Alpha1 (8–10 Hz)	0.18 (0.11–0.32)	0.17 (0.16–0.29)	0.21 (0.12–0.31)	n.s.
Alpha2 (10–13 Hz)	0.09 (0.07–0.13)	0.14 (0.12–0.15)	0.12 (0.09–0.15)	n.s.
Beta (13–30 Hz)	0.16 (0.13–0.22)	0.2 (0.18–0.23)	0.21 (0.17–0.26)	0.03
Median frequency	8.35 (8.17–8.92)	8.99 (8.88–9.26)	8.96 (8.54–9.29)	<0.01*

*AD* patients with Alzheimer’s disease, *MCI-stable* patients with stable or improving cognition over 30 months, *HC* healthy controls, *n.s.* not significant

Data presented are median (interquartile range)

*p < 0.05 by *t* test for AD vs. MCI-stable, representing statistically significant result

#### Connectivity analysis

**Fig. 4:**
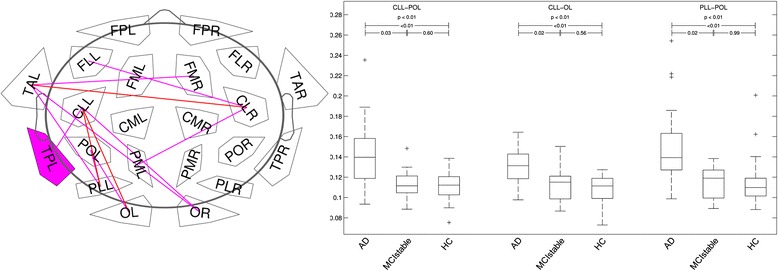
Links (microstate segmented phase lag index [msPLI]) in theta band with significant differences between groups. Plot: *p* < 0.05 (*violet*); *p* < 0.01 (*red*) (corrected for multiple comparisons). Box plot: axes indicate raw msPLI values. *FP* frontal parieto-occipital left, *FM* frontal midline, *FL* frontal lateral, *CM* central midline, *CL* central lateral, *TA* temporal anterior, *TP* temporal posterior, *PL* parietal lateral, *PM* parietal midline, *PO* parieto-occipital, *O* occipital, *L* left, *R* right, *CLL_POL* link centrolateral to parieto-occipital left, *CLL-OL* link centrolateral to occipital left, *PLL-POL* link parietolateral to parieto-occipital left, *AD* Alzheimer’s disease, *MCIstable* patients with stable or improving cognition within 30 months, HC healthy control subjects

**Fig. 5:**
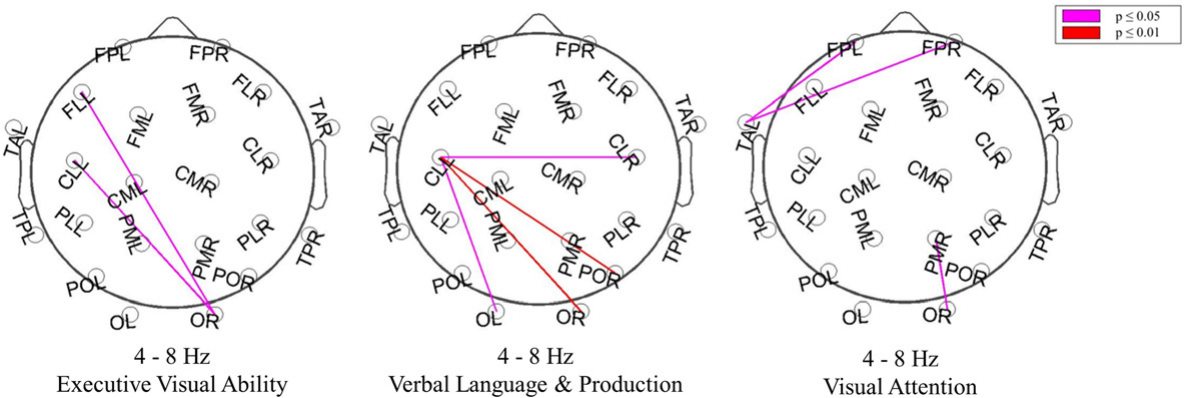
Significant correlations of single links and domain scores. *Violet*: *p* < 0.05; *red*: *p* < 0.01 (corrected for multiple comparisons). *FP* frontal parieto-occipital left, *FM* frontal midline, *FL* frontal lateral, *CM* central midline, *CL* central lateral, *TA* temporal anterior, *TP* temporal posterior, *PL* parietal lateral, *PM* parietal midline, *PO* parieto-occipital, *O* occipital, *L* left, *R* right

After correction for multiple comparisons, no significant differences were found in connectivity calculated without splitting by microstates. Using connectivities from microstates, significant differences in theta-band connectivity were detected (Fig. 4). The theta-band link between the left centrolateral and parieto-occipital regions differentiated most significantly between all three groups and between AD and MCI-stable with a sensitivity of 77 % and a specificity of 78 %. Connectivities were higher in the AD group than in the MCI-stable and HC groups. No significant differences were found for the connectivities in the alpha1, alpha2, and beta bands. Only a few links in the theta band correlated significantly with one of the six domain scores after correction for multiple comparisons. For results, see Fig. 5.

#### Graph analysis

**Fig. 6:**
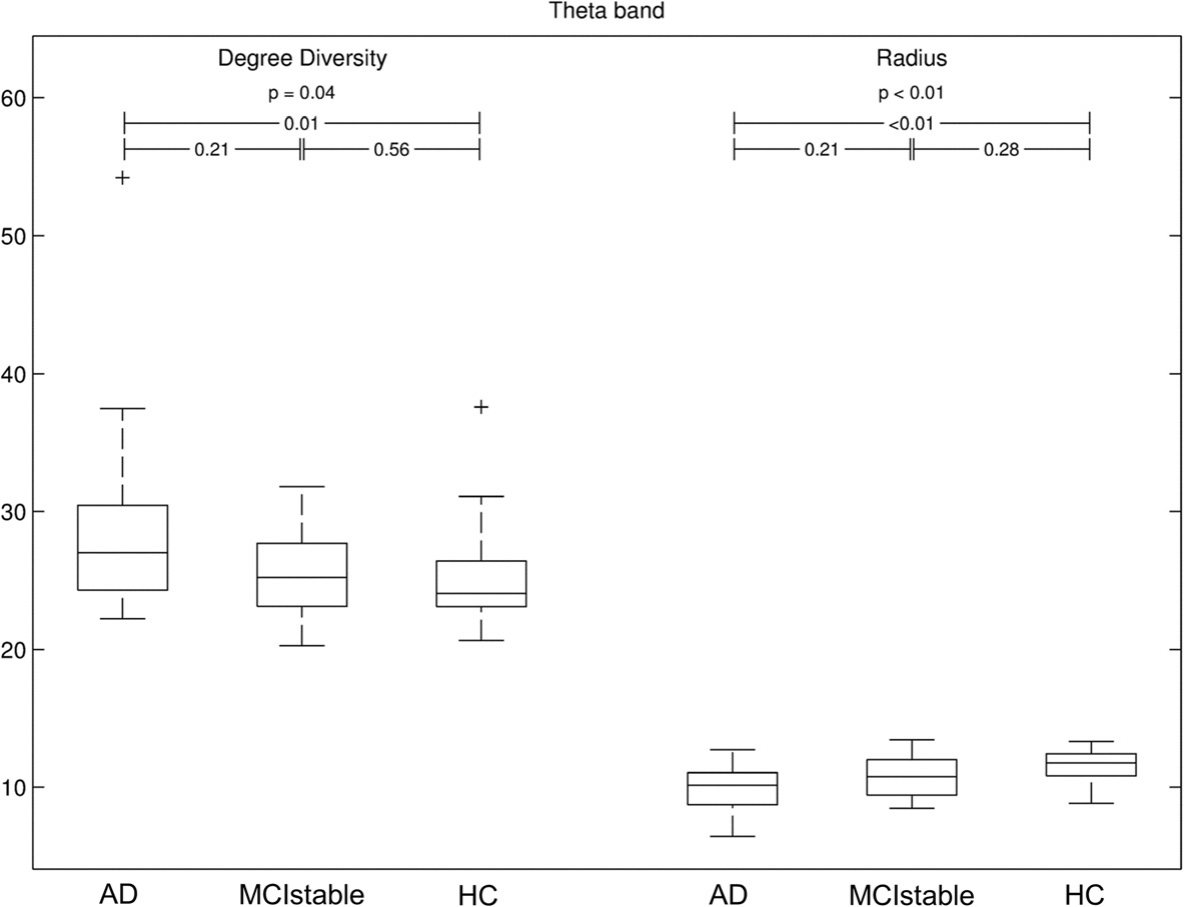
Significant results of graph analysis. Axes indicate raw values of degree diversity and radius *p* values derived by Kruskal-Wallis test for three-group comparisons and Mann-Whitney *U* test for two-group comparisons, '+' are outliers. *AD* Alzheimer’s disease, *MCIstable* patients with stable or improving cognition within 30 months, HC healthy control subjects

Among the results of the graph analysis, only radius of theta connectomes differentiated the three groups. The radius was smaller in the AD group than in the MCI-stable and HC groups. *K*w showed a trend toward higher values in the AD group than in the MCI-stable and HC groups (Fig. 6).

#### Logistic regression model

**Fig. 7:**
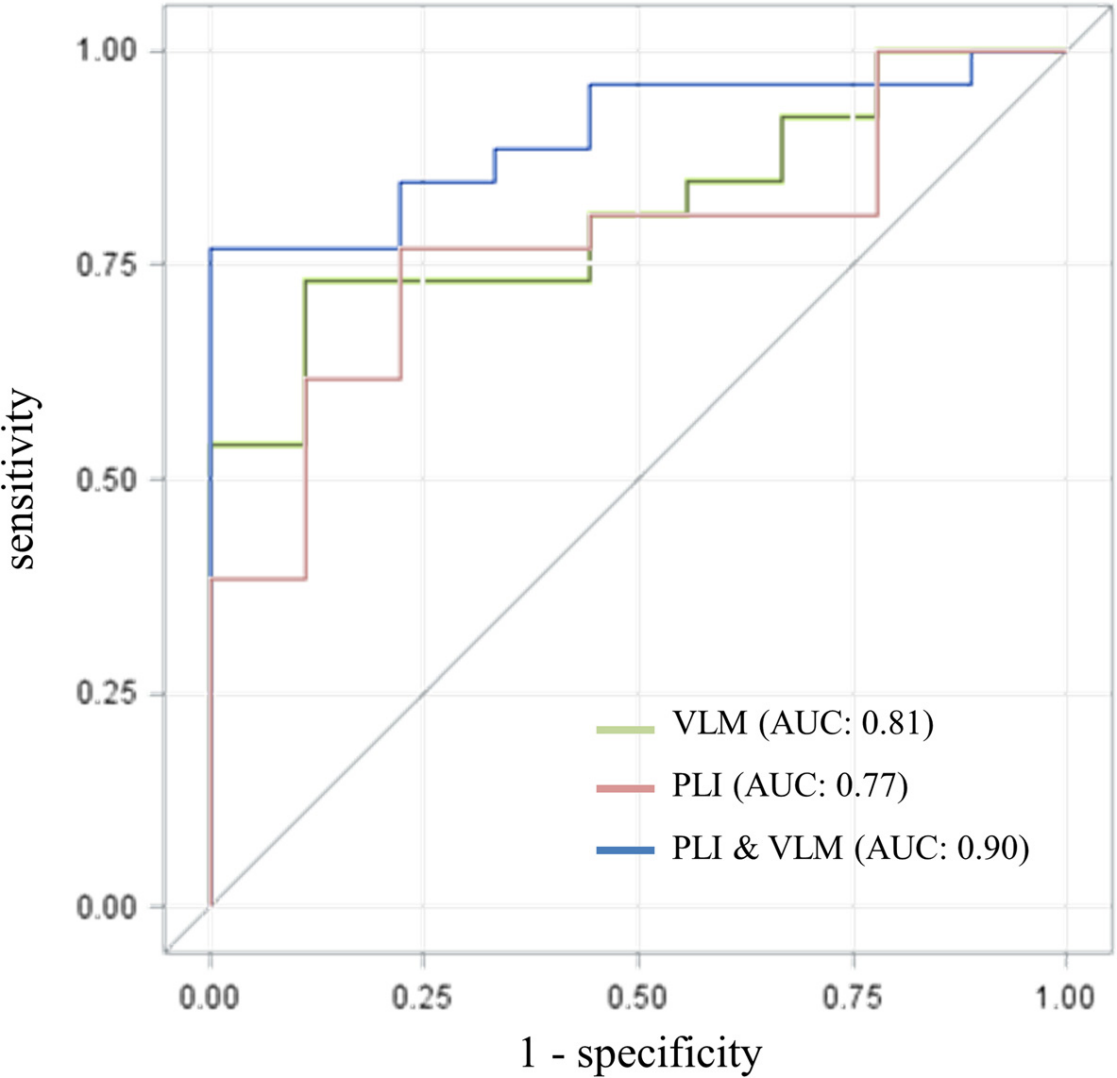
Results of logistic regression comparing Alzheimer’s disease (AD) vs. patients with stable or improving cognition within 30 months (MCI-stable). First, a score was derived using a logistic regression model for the AD and healthy control (HC) groups, then the resulting score was applied to separate the AD group from the MCI-stable group. Score = (10.4 x msPLI) - (3.52 x VLM); a score greater than or equal to −13.2 had sensitivity 76.9 %, specificity 100 %, positive predictive value 100 %, and negative predictive value 60 %. *AUC* area under the curve, *msPLI* microstate segmented phase lag index log of link centrolateral to parieto-occipital left, *VLM* domain score for verbal learning and memory)

A binary logistic regression with the most significant results of the six domain scores at baseline and the frequency and connectivity analysis as well as with AD vs. HC as outcomes was calculated. After stepwise backward elimination, only the connectivity between left centrolateral and parieto-occipital regions (theta band) and verbal learning and memory remained in the model and were used for calculating a score. This score was used in a second logistic regression model in which we compared AD with MCI-stable. The ROC curve showed an area under the curve (AUC) of 0.90 (maximal Youden index sensitivity 77 %, specificity 100 %, positive predictive value 100 %, negative predictive value 60 %) (Fig. 7). Relative theta power was not part of the final model. Adding relative theta power as a third variable increased the AUC only minutely.

## Discussion

A combination of qEEG with neuropsychological measures allows differentiation of patients with early AD from patients with aMCI who remain stable for 30 months with high sensitivity, specificity, and positive predictive value. For differentiation between beginning AD and other patients with MCI, the msPLI seemed to be superior to the PLI. This advantage is most probably explained by an increased signal-to-noise ratio. Microstates are believed to represent activity of different subnetworks of a global network [44, 45]. Using microstate segmentation, EEG periods reflecting the most active subnetworks were selectively included in the analysis, resulting in a more precise estimation of the averaged global network.

Increased msPLI connectivity between the left centrolateral and parieto-occipital regions in the theta band separated AD from MCI-stable with over 90 % positive predictive value and 54 % negative predictive value. Graph analysis in the theta band showed reduced radius and higher *K*w in the AD group compared with the MCI-stable group and even more so compared with the HC group. The combined results of connectivity and graph analysis indicated a shift toward a more hierarchical network with a concentration in a few highly connected nodes, so-called hubs. Hubs are believed to decline disproportionately with disease progression in later stages of AD [46]. In contrast, the present study shows a relative increase of the degree of hubs in very early AD, possibly pointing toward compensatory functional overload leading to faster degradation later. A similar concept was also discussed in a recent article by Morabito et al. [47].

When theta connectivity and verbal learning and memory are combined in a model for predicting the cognitive deterioration of patients with aMCI or early AD, a clear separation between AD and MCI-stable is possible very early after the beginning of cognitive decline. It is not surprising that the domain score for verbal learning and memory remained in the model, as this cognitive domain is most affected in patients with AD [16]; however, the results of relative power analysis, well known to differentiate between groups [12, 30, 48], did not remain in the final model after stepwise backward elimination, as their contribution to the prediction of outcome was included mainly in the two other variables. In contrast, the result for theta connectivity seems to add independent information regarding the 30-month prognosis. The localization of the most affected connections in the parieto-occipital region may be explained by the localization of hubs in the parietal regions, which are known to be particularly affected in AD [46].

Limitations of the study include the small sample size and the low number of patients with mildly reduced yet stable cognition.

## Conclusions

Integration of connectivity results and verbal learning and memory tests in a statistical model may allow for definition of cohorts of patients with MCI with an enhanced risk for AD, a stage at which clinical trials are most promising. The high positive predictive value of this model allows the definition of a patient cohort at great risk of fast cognitive deterioration at a time when they are only mildly affected.

## Additional file

Additional file 1: Figure S1. Mapping of the 22 regions: 170 of 257 electrodes (HydroCel GSN; Electrical Geodesic Inc. [EGI]) were used to define the 22 regions. (TIF 2594 kb)

## Abbreviations

AD: Alzheimer’s disease
aMCI: amnestic mild cognitive impairment
ANOVA: analysis of variance
AUC: area under the curve
CERAD-NAB: Consortium to Establish a Registry for Alzheimer’s Disease Neuropsychological Assessment Battery
*C*w: mean clustering coefficient
EEG: electroencephalography
GFP: global field power
HC: healthy control
*K*w: degree diversity
*L*w: weighted average path length
MCI: mild cognitive impairment
MCI-stable: amnestic mild cognitive impairment patients with stable or improving cognition within 30 months
MMSE: Mini Mental State Examination
msPLI: microstate segmented phase lag index
PLI: phase lag index
qEEG: quantitative electroencephalography
ROC: receiver operating characteristic
*R*w: Pearson correlation of degrees of pairs of neighbors
TAPEEG: Toolbox for Automated Processing of EEG v2.5 software
VLM: verbal learning and memory
