# Supplementary figures and images for: Microstate connectivity alterations in patients with early Alzheimer’s disease
Source: Alzheimers Res Ther. 2015 Dec 31;7:78. doi: 10.1186/s13195-015-0163-9 (PMC4697314; doi:10.1186/s13195-015-0163-9)

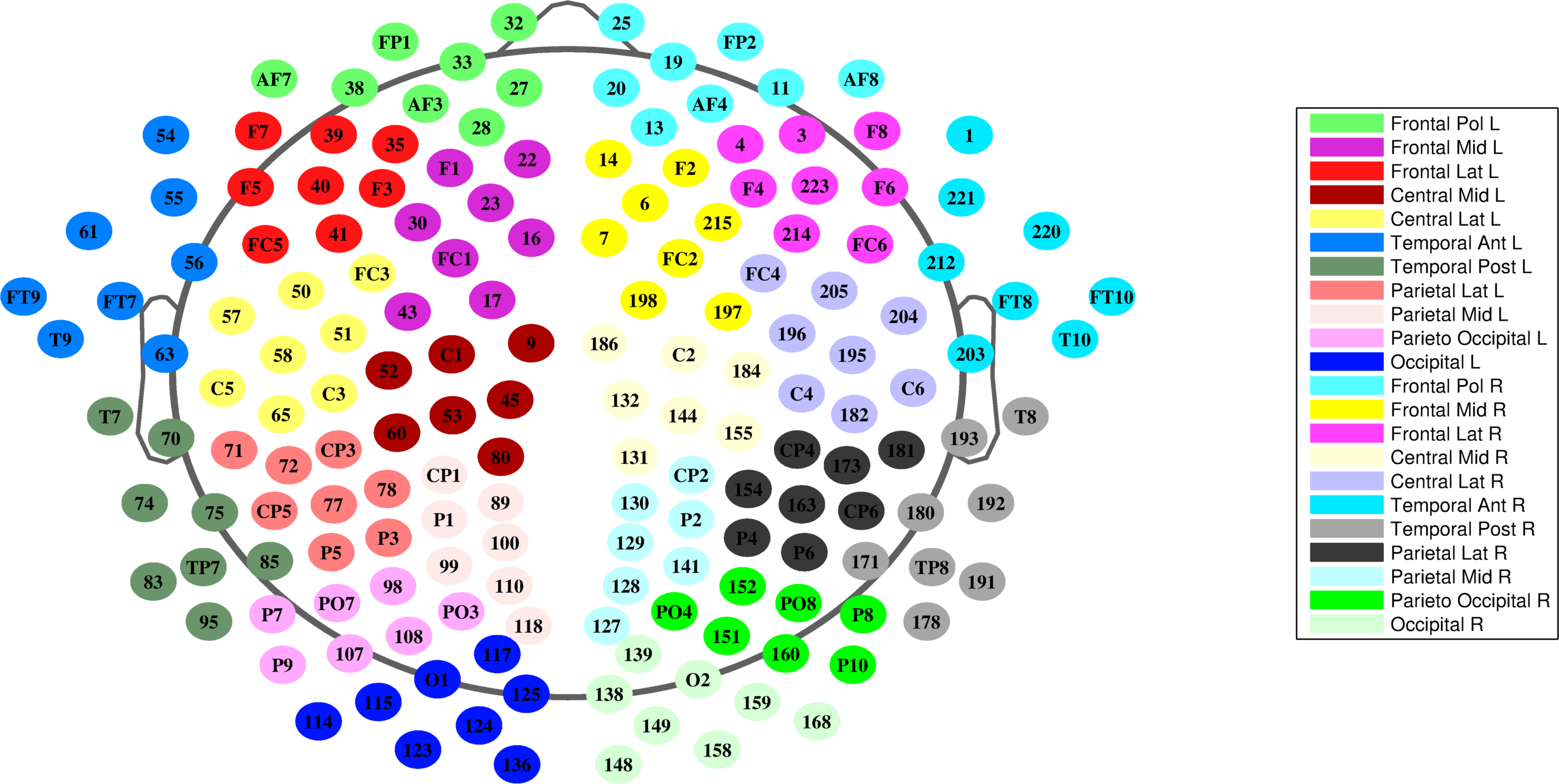

Supplement: Additional file 1: Figure S1. — Mapping of the 22 regions: 170 of 257 electrodes (HydroCel GSN; Electrical Geodesic Inc. [EGI]) were used to define the 22 regions. (TIF 2594 kb) [file 13195_2015_163_MOESM1_ESM.tif]
